# Lysine 27 Ubiquitination of the Mitochondrial Transport Protein Miro Is Dependent on Serine 65 of the Parkin Ubiquitin Ligase*

**DOI:** 10.1074/jbc.M114.563031

**Published:** 2014-03-26

**Authors:** Nicol Birsa, Rosalind Norkett, Tobias Wauer, Tycho E. T. Mevissen, Hsiu-Chuan Wu, Thomas Foltynie, Kailash Bhatia, Warren D. Hirst, David Komander, Helene Plun-Favreau, Josef T. Kittler

**Affiliations:** From the ‡ Department of Neuroscience, Physiology and Pharmacology, University College London, Gower Street, London WC1E 6BT, United Kingdom,; the § Medical Research Council Laboratory of Molecular Biology, Francis Crick Avenue, Cambridge Biomedical Campus, Cambridge CB2 0QH, United Kingdom,; the ¶ University College London Institute of Neurology, Queen Square, London WC1N 3BG, United Kingdom, and; the ‖ Neuroscience Research Unit, Pfizer, Cambridge, Massachusetts 02139

**Keywords:** Mitochondrial Transport, Parkin, Parkinson Disease, Pink1, Ubiquitination, Mitophagy

## Abstract

Mitochondrial transport plays an important role in matching mitochondrial distribution to localized energy production and calcium buffering requirements. Here, we demonstrate that Miro1, an outer mitochondrial membrane (OMM) protein crucial for the regulation of mitochondrial trafficking and distribution, is a substrate of the PINK1/Parkin mitochondrial quality control system in human dopaminergic neuroblastoma cells. Moreover, Miro1 turnover on damaged mitochondria is altered in Parkinson disease (PD) patient-derived fibroblasts containing a pathogenic mutation in the *PARK2* gene (encoding Parkin). By analyzing the kinetics of Miro1 ubiquitination, we further demonstrate that mitochondrial damage triggers rapid (within minutes) and persistent Lys-27-type ubiquitination of Miro1 on the OMM, dependent on PINK1 and Parkin. Proteasomal degradation of Miro1 is then seen on a slower time scale, within 2–3 h of the onset of ubiquitination. We find Miro ubiquitination in dopaminergic neuroblastoma cells is independent of Miro1 phosphorylation at Ser-156 but is dependent on the recently identified Ser-65 residue within Parkin that is phosphorylated by PINK1. Interestingly, we find that Miro1 can stabilize phospho-mutant versions of Parkin on the OMM, suggesting that Miro is also part of a Parkin receptor complex. Moreover, we demonstrate that Ser-65 in Parkin is critical for regulating Miro levels upon mitochondrial damage in rodent cortical neurons. Our results provide new insights into the ubiquitination-dependent regulation of the Miro-mediated mitochondrial transport machinery by PINK1/Parkin and also suggest that disruption of this regulation may be implicated in Parkinson disease pathogenesis.

## Teaser

**Background:** Miro is a mitochondrial protein involved in mitochondrial trafficking.

**Results:** Mitochondrial damage drives rapid Miro ubiquitination in a manner dependent on Ser-65 in Parkin; however, Miro degradation is delayed.

**Conclusion:** Ubiquitination of Miro, rather than its degradation, could act as a signal for mitochondrial arrest and clearance.

**Significance:** Disruption of the mitochondrial transport machinery could be implicated in Parkinson disease.

## Introduction

Mitochondrial transport plays an important role in matching mitochondrial distribution to localized energy demand and calcium buffering requirements. Miro1 and Miro2 are outer mitochondrial membrane (OMM)^8^ proteins that are crucial for regulating mitochondrial trafficking and distribution by coupling mitochondria to the kinesin- and dynein-dependent microtubule transport pathway (1–5). In addition to trafficking, an accurate mitochondrial quality control system is needed to degrade damaged mitochondria that can no longer sustain the cell's metabolic requirements. PINK1 (PTEN-induced putative kinase 1; *PARK6*) and Parkin (*PARK2*) are components of a mitochondrial quality control apparatus that promotes the selective turnover of damaged mitochondria. Loss of function mutations in PINK1 and Parkin are also associated with rare recessive forms of PD (6). PINK1, a mitochondrial serine/threonine kinase normally imported into the mitochondrion and cleaved at the inner mitochondrial membrane, selectively accumulates in its full-length form on the OMM of damaged mitochondria and recruits Parkin, an E3 ubiquitin ligase, from the cytosol to ubiquitinate various OMM substrates (7–12). The ubiquitination by Parkin of OMM proteins is a crucial step in the clearance of damaged mitochondria by mitophagy, and there has been great interest in identifying the mitochondrial Parkin substrates and receptors of this mitochondrial clearance pathway. Several substrates have been identified, including VDAC1, Mitofusins 1 and 2 (Mfn1 and Mfn2), Drp1, Tom20, and Tom40 (13–17) and more recently Miro (10, 18–20). Ubiquitination of Mfn1 leading to its proteasomal degradation may act to sequester damaged mitochondria, whereas ubiquitination of substrates, including VDAC1, is proposed to initiate HDAC6/p62/LC3-dependent autophagosome formation (13, 14, 21). Regulation of the Miro trafficking complex by PINK1 and Parkin may also be critical to block the transport of damaged mitochondria, as removing Miro, the key adaptor that couples mitochondria to the microtubule motor proteins, could isolate the damaged organelles from the functional mitochondrial network (18). However, the mechanisms by which PINK1 and Parkin target and modify Miro leading to its altered function remain unclear. Moreover, how Parkin is anchored to damaged mitochondria, the dynamics, and consequences of Miro ubiquitination and degradation and whether Miro ubiquitination is disrupted in PD related pathology also remain poorly understood.

Here, we characterize in human dopaminergic SH-SY5Y neuroblastoma cells the dynamics of Miro1 ubiquitination by the PINK1/Parkin mitochondrial quality control system. We find that mitochondrial damage triggers rapid (within tens of minutes) and persistent PINK1 and Parkin-dependent Miro ubiquitination, whereas Miro degradation has a much slower onset (hours). Miro ubiquitin chains were found to be predominantly lysine 27 (ubiquitin Lys-27)-mediated, in addition to some Lys-11 and Lys-29, whereas we saw little Lys-48-linked ubiquitination. In SH-SY5Y cells, Miro1 ubiquitination is primarily dependent on Ser-65 within Parkin, a recently reported serine residue at the N terminus of Parkin that is heavily phosphorylated by PINK1 (22). Moreover, we find this site plays an important role in regulating Parkin function on the mitochondrion in cortical neurons. We also show that Miro turnover on damaged mitochondria is altered in PD patient-derived fibroblasts containing a mutation in the *PARK2* gene, suggesting that correct regulation of Miro function may be disrupted in PD. Our results provide new insights into the regulation of the Miro-dependent mitochondrial transport machinery during mitophagy and further suggest that disruption of this regulation may be implicated in PD pathogenesis.

## EXPERIMENTAL PROCEDURES

### 

#### 

##### Constructs

cDNA constructs encoding mtDsRed2, ^GFP^Miro1, and ^myc^Miro1 have been described previously (1, 5, 23). ^GFP^Miro1 S156A and S156E mutations were made by site-directed mutagenesis on the ^GFP^Miro1 backbone; ^GFP^TRAK1 was cloned by insertion of the mouse TRAK1 sequence in the EGFP-C1 vector. The following mutants were from Addgene: pMito-LSSmKate2-N1 (large Stokes shift mKate2) (plasmid 31879 (24)); pRK5-Parkin-Myc (plasmid 17612 (25)); EYFP-Parkin (plasmid 23955 (26)); pRK5-HA-ubiquitin (plasmid 17608 (27)); ubiquitin Lys-27 (plasmid 22902 (28)); Lys-29 (plasmid 22903 (28)); Lys-33 (plasmid 17607 (27)); Lys-48 (plasmid 17605 (27)); Lys-63 (plasmid 17606 (27)); K48R (plasmid 17604 (27)); and K29R (plasmid 17602 (27)). ^HA^Ubiquitin Lys-6, Lys-11, K6R, K11R, K27R, and K27R/K29R mutants were made by site-directed mutagenesis on the pRK5-HA-ubiquitin or on the pRK5-HA-ubiquitin KO (plasmid 17603 (27)) backbone. ^YFP^Parkin S65A and S65E mutations were made by site-directed mutagenesis on the YFP-Parkin backbone. EGFP-C1 was from Clontech. pcDNA4-PINK1-Myc was a gift from Hélène Plun-Favreau, and pSC2-mCherry-Miro1 was a gift from John Carroll.

##### Antibodies and Reagents

Anti-Miro1 (catalog no. HPA010687, recognizing Miro1 and Miro2), anti-Miro2 (catalog no. HPA012624), and anti-TRAK1 antibodies were from Atlas (Miro1 1:1000 and Miro2 and TRAK1 1:500, rabbit); anti-Mfn1 and ApoTrack^TM^ cytochrome *c* Apoptosis WB Antibody Mixture (CVα, PDH E1α, cytochrome *c*, GAPDH) were from Abcam (1:1000, mouse); anti-actin (1:2000, rabbit) and anti-FLAG (1:1000, rabbit) were from Sigma; anti-Tom20 (1:500, rabbit) was from Santa Cruz Biotechnology; anti-mono- and polyubiquitinylated conjugates (FK2) was from Enzo Life Sciences (1:1000, mouse); and anti-GFP antibody was from Neuromab (clone N86/8, 1:100, mouse) or Nacalai Tesque (1:2000, rat). Anti-Myc (9E10) and anti-HA were obtained from 9E10 and 12CA5 hybridoma lines respectively and used as supernatant at 1:100. Secondary HRP-conjugated antibodies were purchased from Rockland and used at 1:5000. Secondary antibodies Alexa-488, Alexa-564, and Alexa-633 were purchased from Invitrogen and used at 1:1000. Dylight-405 was from Jackson ImmunoResearch and used at 1:500.

FCCP (Sigma), was used at 10 μm; MG-132 (Cayman Chemical) was used at 30 μm for 30 min before and during FCCP treatment; bafilomycin 1A (BioVitica) was used at 1 μm for 30 min before and during FCCP treatment; valinomycin (Sigma) was used at 2 μm in Neurobasal medium (Invitrogen) supplemented with 0.6% d-glucose.

##### Cell Culture

^FLAG^Parkin stably overexpressing SH-SY5Y cells are a gift from Dr. Helen Ardley (Leeds Institute of Molecular Medicine) and were previously described (29). PINK1 and nonsilencing shRNA stably expressing SH-SY5Y were previously described (30, 31). SH-SY5Y, COS-7, HeLa, and HEK cells were maintained in Dulbecco's modified Eagle's medium (DMEM, Invitrogen) supplemented with 10% heat-inactivated FBS, penicillin, and streptomycin (2 μg/ml puromycin was added to the SHSY-5Y nonsilencing or PINK1 shRNA-overexpressing cell media as a selection antibiotic). Human fibroblasts were derived from punch biopsies taken with informed consent from a control patient or from a patient carrying a homozygous deletion of exons 3 and 4 of the *PARK2* gene. The ethics for this study were approved by the London-City Road and Hampstead REC committee. The tissue was dissected, and fibroblasts were cultured in DMEM supplemented with 10% heat-inactivated FBS, penicillin, and streptomycin. Cells were kept in 5% CO_2_ at 37 °C, in a humidified incubator.

SH-SY5Y, HeLa, and HEK cells were transfected by nucleofection (Amaxa, Lonza), and COS-7 cells were electroporated using a Capacitance Extender II accessory module (Bio-Rad). Experiments were carried out 48 h post-transfection unless otherwise stated.

Neuronal cultures were prepared from E18 Sprague-Dawley rat embryos of either sex. Briefly, hippocampi and cortices were dissected from E18 embryos. Hippocampi were incubated in 0.125% trypsin and cortices in 0.25% trypsin for 15 min at 37 °C. Neurons were then washed, triturated, and immediately plated on 13-mm coverslips coated with 500 μg/ml poly-l-lysine or on plates coated with 50 μg/ml poly-l-lysine and maintained in Neurobasal plus B27 supplement (Invitrogen). Cortical neurons were transfected by nucleofection before plating as described previously (1, 32, 33), and hippocampal neurons were transfected with Lipofectamine 2000 (Invitrogen) at 10–12 days *in vitro* and analyzed at 13–15 days *in vitro*.

##### Biochemical Assays

For ubiquitination assays, cells were lysed in RIPA buffer (50 mm Tris, pH 7.4, 150 mm NaCl, 1 mm EDTA, 2 mm EGTA, 1% Nonidet P-40, 0.5% deoxycholic acid, 0.1% SDS, 1 mm PMSF, and antipain, leupeptin, pepstatin at 10 μg/ml each). Lysates were then incubated on a rotating wheel at 4 °C for 1 h, and then nuclei and cellular debris were spun down at 20,000 × *g* for 10 min. Supernatants were incubated at 4 °C for 2 h with 10 μl of a 50% slurry of either GFP-TRAP beads (Chromotek) or anti-Myc beads (Sigma). For endogenous Miro1 immunopurification, 0.5 μg of anti-Miro1 was added in parallel with a nonimmune rabbit IgG control antibody for 1.5 h. Complexes were then precipitated for 1.5 h with 15 μl of 50% protein A-Sepharose bead slurry (Generon). Bound material was washed five times in the above buffer before elution with 3× Laemmli sample buffer.

Coimmunoprecipitation experiments were carried out in a similar way. Cells were lysed in 50 mm Tris, pH 7.4, 150 mm NaCl, 1 mm EDTA, 0.5% Triton X-100, 1 mm PMSF, antipain, leupeptin, and pepstatin at 10 μg/ml each. After clearing the lysates of nuclei and cellular debris, the samples were incubated with GFP-TRAP beads; the immunoprecipitated complexes were washed three times with the above buffer and eluted with 3× Laemmli sample buffer. Samples were then western-blotted on nitrocellulose (GE Healthcare), developed with Crescendo substrate (Millipore), and detected with a LAS 4000 imager (GE Healthcare). Densitometric quantification of bands on gels was carried out using the software Quantity One (Bio-Rad).

##### Ub Chain Restriction (UbiCRest) Analysis

UbiCRest assays were performed as described previously (34). Briefly, immunopurified ^GFP^Miro1 was resuspended in 2× deubiquitinase (DUB) reaction buffer (100 mm Tris, pH 7.4, 100 mm NaCl, 10 mm DTT); USP21, OTUD2, and Otulin were diluted in 2× DUB dilution buffer (50 mm Tris, pH 7.4, 300 mm NaCl, 20 mm DTT) and incubated for 10 min at RT. DUBs were then added to the immunoprecipitated ^GFP^Miro1 (on GFP-TRAP beads) and incubated for 30 min at 37 °C. The reaction was stopped by the addition of Laemmli sample buffer; samples were run on a 8–15% SDS-polyacrylamide gel and blotted on PVDF membrane (Amersham Biosciences).

##### Fixed Imaging

For fixed imaging, cells grown on coverslips were washed in 1× PBS and fixed in 4% paraformaldehyde solution (4% paraformaldehyde, 4% sucrose in 1× PBS, pH 7.0) for 15 min. After three further washes in 1× PBS, coverslips were incubated for 10 min in blocking solution (0.5% BSA, 10% horse serum, 0.2% Triton X-100 in 1× PBS). Coverslips were then incubated for 1 h with primary antibodies diluted in blocking solution, washed five times in 1× PBS, and incubated with secondary antibodies. After a further five washes in 1× PBS coverslips were mounted on glass slides using ProGold anti fade reagent (Invitrogen) and later sealed with nail varnish.

Imaging was carried out using a Zeiss LSM 700 upright confocal microscope with a 63× oil-immersion lens with 1.4 numerical aperture. Images were digitally captured using ZEN 2010 software.

##### Image Processing

Images were processed in ImageJ or Metamorph. For colocalization studies, images were taken with comparable Hi/Low ratio and then arbitrarily thresholded. Integrated colocalization function (Metamorph) was used to measure the colocalization. In neurons, Parkin and ubiquitin translocation to mitochondria were scored manually on blinded samples, using the Cell Counter plug-in for ImageJ.

##### Statistical Analysis

All data were obtained using cells from at least three different preparations. The numbers of cells studied are given in the text or figure legends. Statistical significance across groups was analyzed using one-way analysis of variance and Dunnett's post hoc test to compare the data groups with their reference group or the Bonferroni's post hoc test to compare all data groups. Individual differences were assessed using individual Student's *t* tests. Data are shown as mean ± S.E.

## RESULTS

### 

#### 

##### PINK1 and Parkin Regulate the Loss of Miro1 and Miro2 upon Mitochondrial Damage in Human Dopaminergic Neuroblastoma Cells and Fibroblasts

**FIGURE 1.**
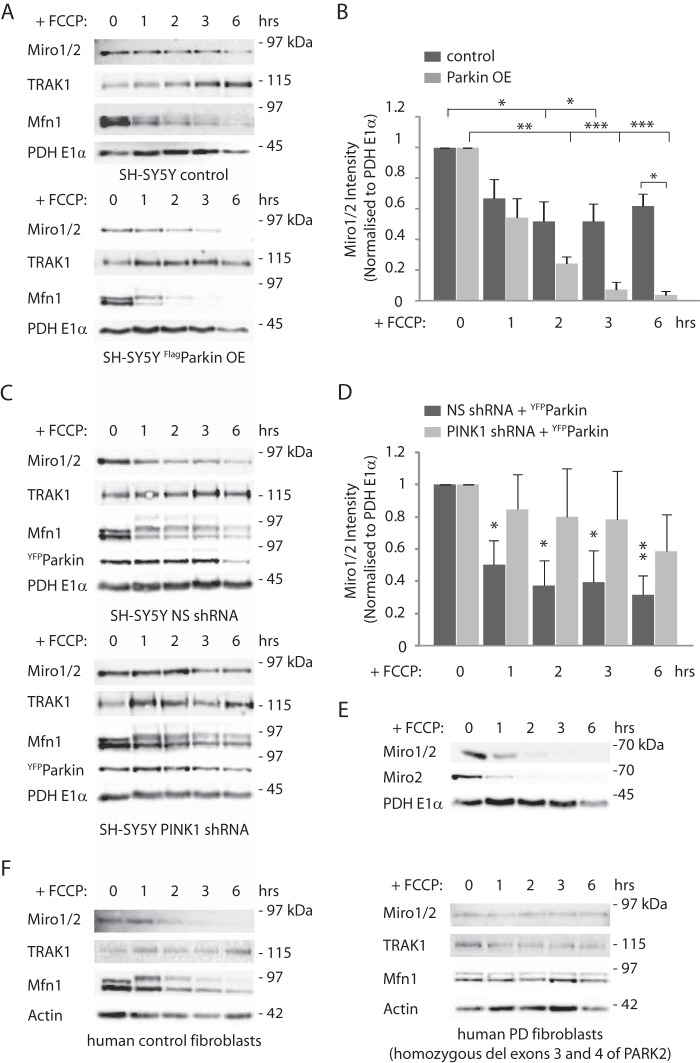
**Mitochondrial damage triggers PINK1/Parkin-dependent Miro1/2 degradation in a dopaminergic neuroblastoma cell line.** *A* and *B,* control SH-SY5Y cells or stably overexpressing ^FLAG^Parkin SH-SY5Y (^FLAG^Parkin OE) cells were incubated with FCCP (10 μm) for the indicated time. *A,* FCCP time course experiment indicating the loss of the outer membrane proteins Miro1/2 and Mfn1 but not of the trafficking adaptor TRAK1 or the mitochondrial matrix protein PDH E1α. Loss of Miro1/2 and Mfn1 is favored by Parkin overexpression. *B, bar graph* quantifying the FCCP-dependent loss of Miro1/2. Miro1/2 normalized mean intensity in control SH-SY5Y cells at 1 h FCCP 0.67 ± 0.12 S.E.; 2 h, 0.52 ± 0.13 S.E.; 3 h, 0.52 ± 0.12 S.E., and 6 h, 0.62 ± 0.08 S.E.; *n* = 6. Miro1/2 mean intensity in ^FLAG^Parkin OE SH-SY5Y cells at 1 h FCCP 0.54 ± 0.12 S.E.; 2 h, 0.24 ± 0.04 S.E.; 3 h, 0.07 ± 0.05; 6 h, 0.04 ± 0.02 S.E.; *n* = 3, *, *p* < 0.05; **, *p* < 0.01; ***, *p* < 0.001. *C* and *D,* SH-SY5Y cells stably expressing either nonsilencing (*NS*) shRNA or PINK1 shRNA and transfected with ^YFP^Parkin were treated with FCCP (10 μm) for the indicated time points. *C,* representative western blot of a FCCP time course. Miro1/2 loss is inhibited by the specific knockdown of PINK1. *D, bar graph* quantifying this effect. Miro1/2 normalized mean intensity in nonsilencing shRNAi at 1 h FCCP, 0.50 ± 0.15 S.E.; 2 h, FCCP 0.37 ± 0.16 S.E.; 3 h, FCCP 0.39 ± 0.20 S.E., and 6 h, FCCP 0.31 ± 0.12 S.E.; Miro1/2 mean intensity in PINK1 shRNAi expressing cells at 1 h FCCP 0.84 ± 0.22 S.E.; 2 h, FCCP 0.80 ± 0.30 S.E.; 3 h, FCCP 0.78 ± 0.30 S.E., and 6 h, FCCP 0.58 ± 0.23 S.E.; *n* = 4; significance is calculated comparing treated samples to control (0 h) *, *p* < 0.05; **, *p* < 0.01. *E,* SH-SY5Y cells stably overexpressing ^FLAG^Parkin were treated with FCCP (10 μm) for several time points. Miro1/2 and Miro2 show similar degradation kinetics, *n* = 3. *F,* FCCP treatment (10 μm) of human control fibroblasts triggers the loss of Miro1/2 as well as Mfn1 (*left panel*); however, FCCP-induced mitochondrial damage in human fibroblasts derived from a PD patient carrying a deletion of exons 3 and 4 of the *PARK2* gene does not cause loss of Miro1/2 or Mfn1 (*right panel*).

To identify and further characterize PINK1- and Parkin-mediated regulation of Miro proteins in neuronal cells, human-derived dopaminergic SH-SY5Y neuroblastoma cells, where Parkin has been previously shown to undergo mitochondrial translocation upon mitochondrial damage (13), were analyzed using western blotting. Exposure of SH-SY5Y cells to the mitochondrial uncoupler FCCP led to a loss of endogenous Miro1/2 protein at its expected molecular weight (detected with an anti-Miro1 antibody that cross-reacts with Miro2, data not shown) within 2 h of FCCP treatment (Fig. 1, *A, top panel,* and *B*), similar to the loss of Miro1 previously reported upon induction of mitochondrial damage in HEK and HeLa cells (18, 19), whereas the mitochondrial matrix protein PDH E1α was unaffected. To further examine the role of Parkin in this process, we evaluated the kinetics of Miro1/2 loss in control SH-SY5Y cells compared with SH-SY5Y cells constitutively overexpressing Parkin (Parkin OE) and that exhibit enhanced mitophagy. A significant facilitation of Miro1/2 loss upon FCCP-induced mitochondrial damage could be observed in the Parkin OE cells compared with control (Fig. 1, *A, bottom panel,* and *B*). To further determine whether there was a substantial difference in FCCP-induced loss of Miro1 *versus* Miro2, we tested the specific loss of Miro2 in Parkin OE cells using a Miro2-specific antibody, which revealed that endogenous Miro1 and Miro2 appear to be lost with similar kinetics (Fig. 1*E*, *n* = 3). PINK1 acts upstream of Parkin to recruit the ligase to the OMM upon mitochondrial damage (35). To investigate whether PINK1 was involved in damage-induced Miro loss, we used previously characterized SH-SY5Y cells, expressing PINK1 shRNA or a nonsilencing shRNA (30, 31). FCCP treatment induced a decrease in Miro1/2 levels in nonsilencing shRNA expressing SH-SY5Y cells transfected with ^YFP^Parkin; however, this effect was inhibited in SH-SY5Y cells expressing PINK1 RNAi and transfected with ^YFP^Parkin (Fig. 1, *C* and *D*).

We also monitored the stability of other components of Miro protein complexes, including the Miro-binding kinesin adaptor TRAK1 (2, 36, 37) and the mitochondrial fusion GTPase Mfn1, a key regulator of mitochondrial dynamics, which also interacts with Miro (38). FCCP-induced Mfn1 loss was observed to rapidly occur upon mitochondrial damage facilitated by Parkin overexpression and blocked by PINK1 knockdown similar to previous reports (15, 39), although we did not observe a significant change in TRAK1 protein levels under these conditions (Fig. 1, *A* and *C*).

To further investigate the impact on Miro regulation in pathological conditions, we also assessed by western blotting the Parkin-dependent loss of Miro1/2 upon mitochondrial damage in human patient-derived fibroblasts carrying a homozygous deletion of exons 3 and 4 of the *PARK2* gene encoding Parkin. Compared with control fibroblasts, where FCCP induced a loss of Miro1/2 in Parkin-deficient fibroblasts, Miro1/2 levels remained stable (Fig. 1*F*). Thus, Miro levels upon mitochondrial damage, in dopaminergic neuroblastoma cells and in human fibroblasts, are controlled by the PINK1/Parkin pathway. Moreover, the regulation of Miro levels is disrupted in fibroblasts with impaired Parkin function derived from a patient with PD.

##### Ubiquitination of Miro by a PINK1 and Parkin Complex upon Mitochondrial Damage

**FIGURE 2.**
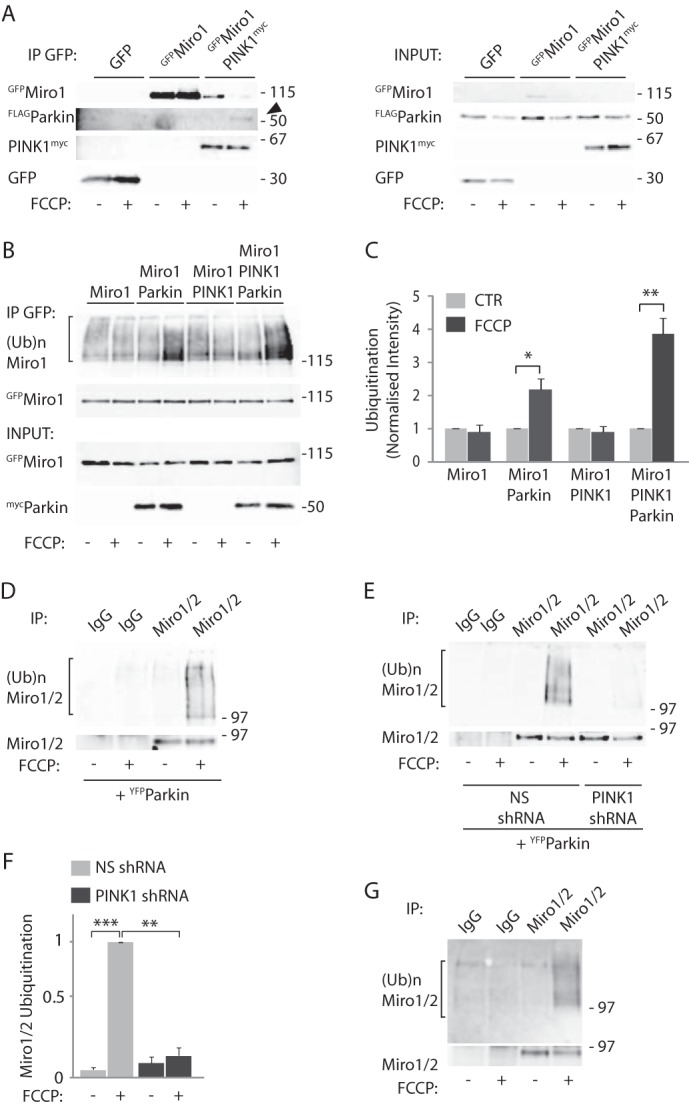
**GTPase Miro1 interacts with and is ubiquitinated by the PINK1-Parkin complex.** *A,* coimmunoprecipitation experiments carried out in SH-SY5Y cells stably overexpressing ^FLAG^Parkin and transfected with GFP or ^GFP^Miro1 and PINK1^myc^ show that Miro1 interacts with PINK1, and mitochondrial depolarization (10 μm FCCP, 1 h) triggers the formation of a complex with Parkin. *B* and *C,* ubiquitination assays demonstrate that FCCP (10 μm, 1 h) triggers Parkin-dependent ubiquitination of exogenous ^GFP^Miro1 in COS-7 cells, detected as a higher molecular weight smear of the immunopurified protein (amount of ubiquitinated Miro1 upon FCCP treatment in control cells 0.88 ± 0.23 S.E., in Parkin OE cells 2.17 ± 0.33 S.E., in PINK1 OE cells 0.88 ± 0.18 S.E., in PINK1 and Parkin OE cells 3.85 ± 0.48 S.E.; *, *p* = 0.027; **, *p* = 0.006, *n* = 4). *D,* FCCP triggers the ubiquitination of endogenous Miro1/2 in the neuroblastoma SH-SY5Y cell line, *n* = 3. *E* and *F,* this process is inhibited by the stable expression of a PINK1 RNAi (ubiquitinated Miro mean intensity in PINK1 shRNA cells 0.16 ± 0.07 S.E. compared with ubiquitinated Miro1 intensity in nonsilencing shRNA expressing cells 1.0; *n* = 3, **, *p* = 0.006; ***, *p* = 0.0008). *G,* ubiquitination of endogenous Miro1/2 can be detected with the endogenous ubiquitin antibody FK2, *n* = 3. *IP*, immunoprecipitation.

Our results support a key role for the PINK1/Parkin pathway in regulating Miro levels upon mitochondrial damage in human dopaminergic SH-SY5Y cells and fibroblasts. In agreement with this, using coimmunoprecipitation assays in Parkin OE SH-SY5Y cells heterologously coexpressing GFP-tagged Miro1 (^GFP^Miro1) with Myc-tagged PINK1 (PINK1^myc^), we could readily demonstrate the interaction between Miro and PINK1, as reported previously in HEK cells (18, 19, 40). Moreover, in agreement with Parkin being recruited to the mitochondrial membrane only upon mitochondrial damage, we found that the formation of a complex between Miro and Parkin could also be observed, but this was only detected upon FCCP-induced damage (Fig. 2*A*).

**FIGURE 3.**
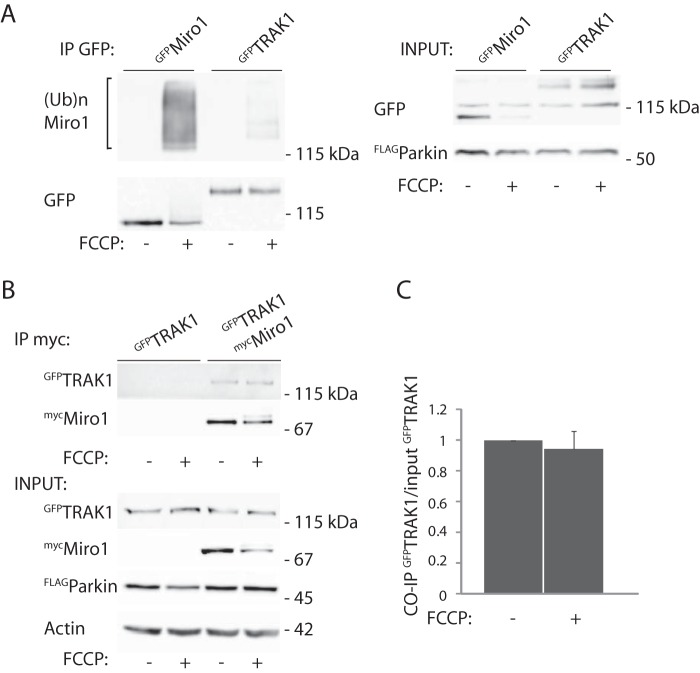
**Effect of mitochondrial damage on TRAK1 ubiquitination and its interaction with Miro1.** *A,* ubiquitination assays performed in SH-SY5Y cells stably overexpressing ^FLAG^Parkin and transfected with either ^GFP^Miro1 or ^GFP^TRAK1. FCCP treatment (10 μm, 1 h) triggers little ubiquitination of TRAK1 compared with Miro1; *n* = 3. *B,* coimmunopurification assay shows no change in the interaction between ^myc^Miro1 and ^GFP^TRAK1 upon mitochondrial damage. *C,* quantification of this effect (co-IP TRAK1 with FCCP 0.94 ± 0.11, compared to control 1); *n* = 3. *IP*, immunoprecipitation.

The loss of Miro1/2 at its expected molecular weight upon mitochondrial damage is likely due to Miro ubiquitination by Parkin. Indeed, we observed a robust increase in the levels of ^GFP^Miro1 ubiquitination, which could be seen as a high molecular weight smear in the ^GFP^Miro1 immunoprecipitates, upon 1 h of FCCP treatment in COS-7 cells overexpressing Parkin alone but not PINK1 alone. Coexpression of Parkin together with PINK1 led to a further significant potentiation of ^GFP^Miro1 ubiquitination (Fig. 2, *B* and *C*). ^GFP^Miro1, and to a lesser extent ^GFP^Miro2 (data not shown), were also found to be robustly ubiquitinated in SH-SY5Y cells upon mitochondrial damage, whereas the Miro and kinesin adaptor TRAK1 exhibited very little ubiquitination (Fig. 3*A*) in agreement with its relatively stable expression during the FCCP damage time course (Fig. 1). Importantly, FCCP-induced ubiquitination of endogenous Miro1/2 could also be observed in neuroblastoma cells overexpressing ^YFP^Parkin (Fig. 2*D*) and was dependent on PINK1 because ubiquitination levels were greatly reduced in dopaminergic SH-SY5Y cells expressing shRNA to PINK1 compared with nonsilencing shRNA-expressing cells (Fig. 2, *E* and *F*). We also confirmed FCCP-induced ubiquitination of endogenous Miro1/2 using antibodies to endogenous mono- and polyubiquitin (Fig. 2*G*).

To test whether Miro1 ubiquitination could affect its ability to interact with the mitochondrial motor complex, we analyzed Miro1 binding to the kinesin adaptor TRAK1 by coimmunoprecipitation; however, in our conditions mitochondrial damage (10 μm FCCP, 1 h) does not affect this interaction (Fig. 3, *B* and *C*).

##### Lys-27-type Ubiquitination of Miro by a PINK1 and Parkin Complex in Dopaminergic Neuroblastoma Cells

**FIGURE 4.**
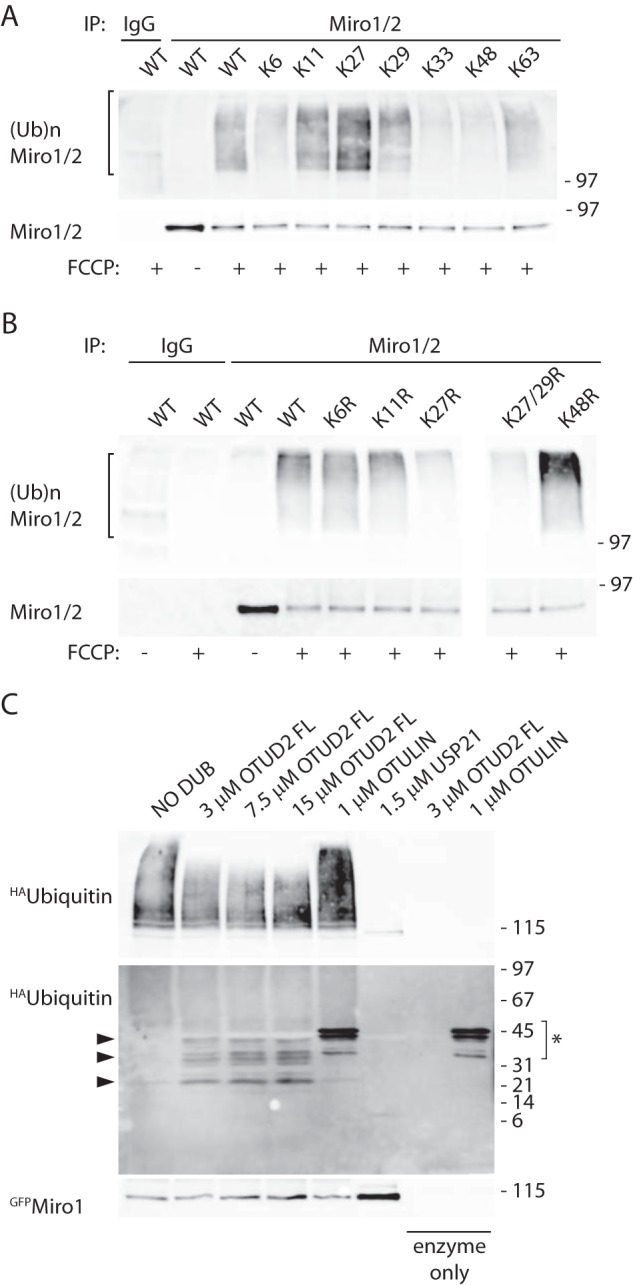
**Analysis of Miro1 ubiquitin chains.** *A,* ubiquitination assays performed in SH-SY5Y cells stably overexpressing ^FLAG^Parkin and transfected with either wild type ^HA^ubiquitin or several ubiquitin mutants that allow only specific chain types (Lys-6, Lys-11, Lys-27, Lys-29, Lys-33, Lys-48, and Lys-63) reveal that Miro1/2 is primarily ubiquitinated in a Lys-27-dependent manner; *n* = 3. *B,* same assay is carried out using ^HA^ubiquitin mutants that cannot mediate specific ubiquitin chain linkages (K6R, K11R, K27R, K27R/K29R, K48R; *n* = 3). *C,* immunopurified ^GFP^Miro1 was incubated with increasing amounts of OTUD2 (that specifically cleaves Lys-11, Lys-27, Lys-29, and Lys-33, mediated ubiquitin linkages), Otulin (cleaves Met-linked chains) or USP21 (chain type aspecific DUB). *Black arrowheads* indicate HA-positive bands, likely to be ubiquitin trimers and dimers; *asterisk* indicates cross-reactivity between Otulin and the anti-HA antibody. *IP*, immunoprecipitation.

Because the linkage type of the ubiquitin chain can determine the cellular outcome of ubiquitination (41), we explored which chain type(s) were assembled by Parkin on Miro1. To this end, HA-tagged ubiquitin mutants permissive for only one type of ubiquitin chain were expressed in Parkin OE SH-SY5Y cells. Strikingly, immunopurification of endogenous Miro1/2 from these cell lysates revealed a high level of Lys-27 ubiquitin linkage on Miro1/2 (in addition to some Lys-11, Lys-29, and Lys-63 linkages, Fig. 4*A*). In contrast, little Lys-48 ubiquitination was observed, and consistent with this, in cells expressing a ubiquitin mutant where Lys-48 was mutated to arginine (K48R) to block Lys-48 ubiquitination, there was still substantial Miro1 ubiquitination (Fig. 4*B*). This is consistent with previous reports demonstrating accumulation of Lys-27-linked ubiquitin chains on mitochondria after FCCP treatment and Lys-27 polyubiquitination of VDAC1 (13). Implication of Lys-27 linkage was also confirmed by a reduction of Miro1/2 ubiquitination smear with K27R and K27R/K29R ubiquitin mutants (Fig. 4*B*). To further investigate Miro1 ubiquitin chain composition without affecting the chain formation by introducing ubiquitin mutants, we have used chain-specific DUBs in ubiquitin chain restriction (UbiCRest) analysis (42). For this, immunoprecipitated ^GFP^Miro1 was incubated with several DUBs of distinct specificities. USP21 is a highly active, ubiquitin-specific but linkage-aspecific DUB (43). USP21 efficiently cleaves ^GFP^Miro1 ubiquitin chains, generating unmodified ^GFP^Miro1, mono-ubiquitin, and a small amount of mono-ubiquitinated ^GFP^Miro1 (Fig. 4*C*). This confirms that the HA-positive Miro1 smear is exclusively polyubiquitinated ^GFP^Miro1.

An enzyme that specifically recognizes and cleaves Lys-27 linkages has not been identified to date. However, OTUD2 shows linkage preference for Lys-11, Lys-27, Lys-29, and Lys-33 linkages and is the only human ovarian tumor DUB cleaving Lys-27 chains efficiently. Incubation of ^GFP^Miro1 with increasing concentrations of OTUD2 results in a progressive shortening of high molecular weight ^GFP^Miro1 ubiquitination, partial rescue of ^GFP^Miro1 at its expected molecular weight, and the formation of HA-positive bands at 20 and 30 kDa (Fig. 4*C, black arrowheads*), which are likely to be ubiquitin dimers or trimers. The latter could be released from mixed linkage chains on ^GFP^Miro1. In contrast, incubation of ^GFP^Miro1 with the highly active but Met-1/linear chain-specific DUB Otulin (44) does not affect ^GFP^Miro1 ubiquitination (Fig. 4*C*). This confirms that ^GFP^Miro1 comprises atypical polyubiquitin chain linkages, likely Lys-27 chains in addition to some Lys-11 and Lys-29.

##### Kinetics of Miro Ubiquitination and Degradation

**FIGURE 5.**
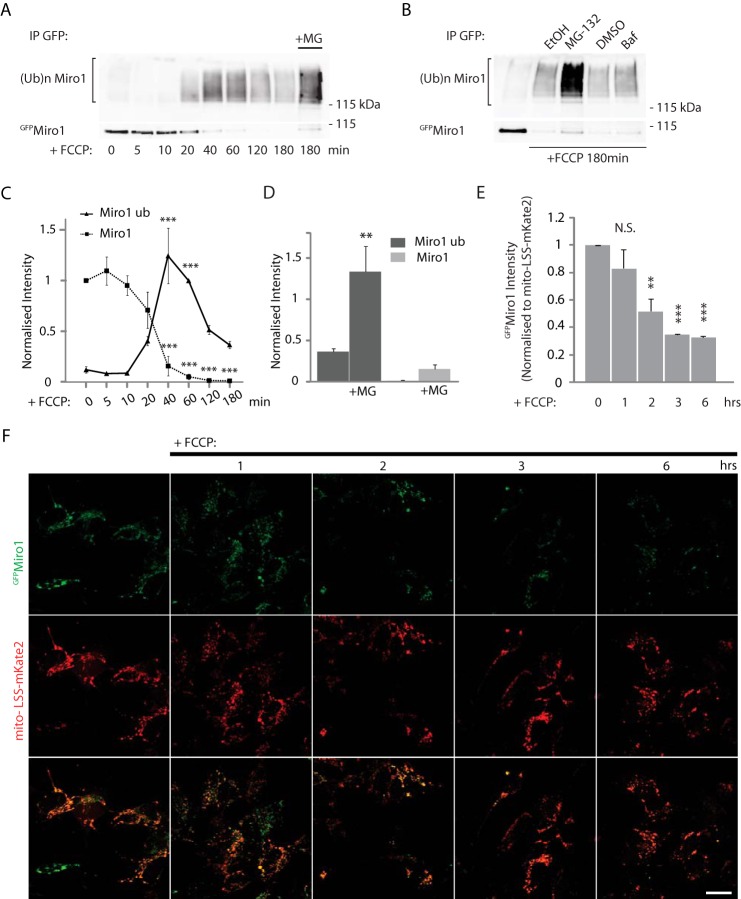
**Temporal dynamics of Miro1 ubiquitination and degradation.** *A,* ^FLAG^Parkin stably overexpressing SH-SY5Y cells were incubated with FCCP (10 μm) for several time points before ubiquitination assays were performed. ^GFP^Miro1 ubiquitination can be detected after 20 min of incubation with the mitochondrial uncoupler (FCCP) and peaks after 40–60 min. Although the ^GFP^Miro1 band disappears at 60 min, an actual decrease in the higher molecular weight species (ubiquitinated ^GFP^Miro1) is detected only after 120 min. *B,* degradation is blocked by pretreatment with the proteasomal blocker MG-132 but not by the lysosomal inhibitor bafilomycin 1A. *C, graph* quantifying Miro1 bands (immunoprecipitated, *dashed line*) and ubiquitination smear intensity in *A*. Average intensities of Miro1 (immunoprecipitated (*IP*)) are normalized to Miro1 (*IP*) at 0 min, whereas Miro1 ubiquitination smear intensities are normalized to the smear intensity at 60 min. Mean intensity of ubiquitinated ^GFP^Miro1 at 0 min, 0.12 ± 0.08; 5 min, 0.08 ± 0.03; 10 min, 0.09 ± 0.04; 20 min, 0.41 ± 0.09; 40 min 1.24 ± 0.23, 60 min 1; 120 min 0.52 ± 0.09; and 180 min 0.37 ± 0.08, *n* = 3, ***, *p* < 0.005. Data are shown as mean ± S.E. *D, bar graph* quantifying the effect of MG-132 in *A*, mean intensity of ^GFP^Miro1 ubiquitination at 180 min with MG-132 1.33 ± 0.24 compared with 0.37 ± 0.08 at 180 min without MG-132, *n* = 3, **, *p* = 0.006. *E* and *F,* ^FLAG^Parkin overexpressing SH-SY5Y cells co-transfected with ^GFP^Miro1 and the mitochondrial marker mito-LSS-mKate2. Confocal imaging of FCCP time course experiment shows a significant decrease of ^GFP^Miro1 signal starting at 2 h, which is consistent with the biochemical data. *E,* quantification of ^GFP^Miro1 intensity normalized to LSS-mito-mKate2 signal. Normalized ^GFP^Miro1 intensity at 0 h, 1.0; 1 h, 0.83 ± 0.14; 2 h, 0.52 ± 0.09; 3 h, 0.35 ± 0.003; and at 6 h, 0.33 ± 0.01, *n* = 3, **, *p* < 0.01; ***, *p* < 0.005; *scale bar,* 20 μm. N.S. not significant. Data are shown as mean ± S.E.

Ubiquitination of Miro could lead to its rapid loss from the OMM by degradative pathways, or alternatively Miro could remain on the mitochondrial membrane in a ubiquitinated form where it may act in another ubiquitination-mediated process such as signaling. To better understand the functional consequences of Miro regulation by the PINK1/Parkin pathway, we further investigated the kinetics of Miro1 ubiquitination, performing a time course of FCCP-induced mitochondrial damage in dopaminergic Parkin OE SH-SY5Y cells. We found that the kinetics of the FCCP-dependent increase of ubiquitinated ^GFP^Miro1 correlated closely with those of the loss of immunoprecipitated ^GFP^Miro1 at 100 kDa. FCCP-induced loss of unmodified ^GFP^Miro1 at its expected molecular mass (100 kDa) could be seen within as little as 20 min. In parallel, levels of ubiquitinated (high molecular weight smear) ^GFP^Miro1 rapidly increased with a similar time course and appeared to remain stable for at least 1 h. With slower kinetics, ubiquitinated ^GFP^Miro1 then appears to undergo degradation, which can be observed by a clear decrease in the levels of ubiquitinated high molecular weight smear ^GFP^Miro1 within the 2nd h of FCCP treatment (Fig. 5, *A* and *C*). This delayed degradation of the ubiquitinated ^GFP^Miro1 appears to be dependent on the activity of the proteasome. In fact, the loss of ubiquitinated ^GFP^Miro1 at later time points could be blocked by proteasomal inhibition with MG-132, but not by lysosomal inhibition with bafilomycin 1A (Fig. 5, *A, B* and *D*).

To further investigate Miro stability on the OMM, we quantified ^GFP^Miro1 levels on the mitochondria using confocal microscopy, normalizing levels of ^GFP^Miro1 to the mitochondrial marker mito-LSS-mKate2. In agreement with our biochemical results, we found that ^GFP^Miro1 levels remained stable on the OMM for at least 60 min after induction of mitochondrial damage with FCCP and well beyond the initial fast ubiquitination, as can be observed by the relatively stable levels of ^GFP^Miro1 fluorescence signal. Only at later stages (after 2 h) did ^GFP^Miro1 fluorescence intensity begin to decrease, correlating closely with the observed delayed degradation of ubiquitinated Miro (Fig. 5, *E* and *F*). These results demonstrate that in human dopaminergic neuroblastoma cells Miro is initially rapidly ubiquitinated on the OMM where it appears to remain in a ubiquitinated form before undergoing a slower degradation and removal, dependent on proteasomal activity.

##### Role of Miro and Parkin Phosphorylation in Regulating Miro Ubiquitination and Degradation

**FIGURE 6.**
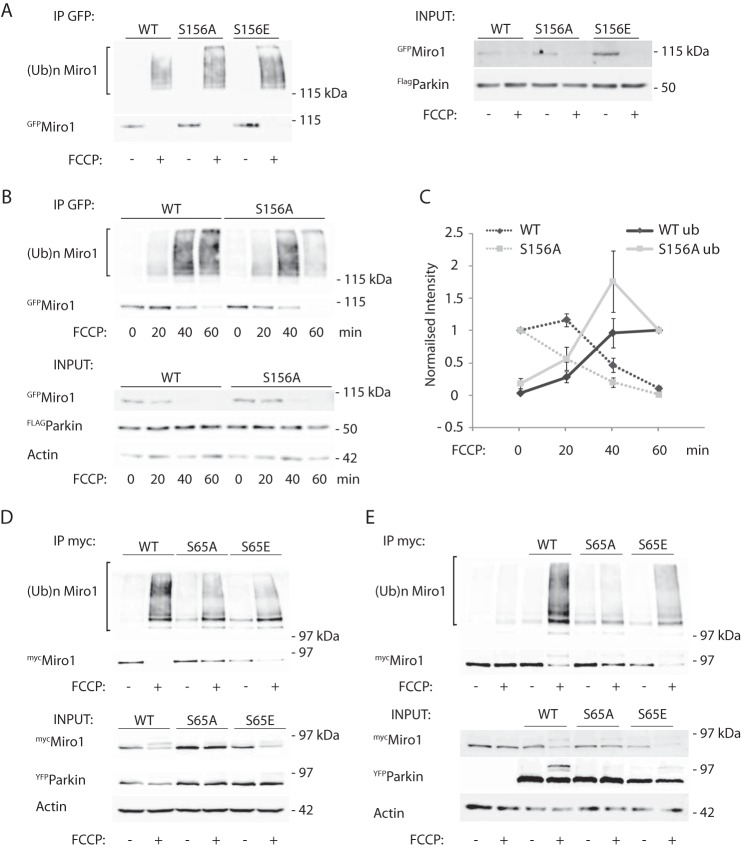
**Miro1 phosphorylation does not affect its ubiquitination dynamics, whereas Parkin phosphorylation does.** *A–C,* ^GFP^Miro1 phospho-null (S156A) or phospho-mimetic (S156E) mutants show no difference in ubiquitination dynamics compared with wild type Miro1 in Parkin OE SH-SY5Y cells; *C,* in ubiquitinated S156A Miro1 mean intensity at 0 min, 0.19 ± 0.08; at 20 min, 0.57 ± 0.17; at 40 min, 1.75 ± 0.47; at 60 min, 1.0, ubiquitinated wild type Miro1 mean intensity at 0 min, 0.04 ± 0.004; at 20 min, 0.29 ± 0.08; at 40 min, 0.96 ± 0.22; at 60 min, 1.0; *n* = 3. Data are shown as mean ± S.E. *D,* expression of the phospho-null ^YFP^Parkin S65A mutant in HeLa cells blocks FCCP-induced ^myc^Miro1 ubiquitination and its subsequent loss. Phospho-mimetic S65E Parkin allows a residual Miro1 ubiquitination; *n* = 3. *E,* same effect is observed in SH-SY5Y dopaminergic cells; *n* = 1.

Several reports have suggested that phosphorylation of OMM proteins by PINK1 is a critical step in the initiation of mitophagy. A previous report showed that phosphorylation of Miro1 on serine 156 by PINK1 was important for Parkin-mediated control of Miro levels upon mitochondrial damage in HEK and HeLa cells (18). We therefore investigated whether phosphorylation of Miro by PINK1 could similarly regulate the dynamics of its ubiquitination by Parkin in dopaminergic SHSY-5Y cells. To address the role of Miro1 phosphorylation for regulating the ubiquitination of Miro in dopaminergic neuroblastoma cells, we tested the ability of phospho-null (S156A) and phospho-mimetic (S156E) mutants of Miro1 to be ubiquitinated upon mitochondrial damage. In contrast to previous observations made in HEK and HeLa cells (18), we found no major influence of this Miro1 phosphorylation site on Miro1 expression levels or ubiquitination levels upon mitochondrial damage in Parkin OE SH-SY5Y cells (Fig. 6*A*, *n* = 3). We further investigated the kinetics of Miro1 ubiquitination upon a time course of FCCP-induced mitochondrial damage in the Miro phospho-null (S156A) mutant (Fig. 6*B*), but we found that in SH-SY5Y cells the phospho-null mutant could undergo ubiquitination with kinetics similar to that of wild type Miro (Fig. 6, *B* and *C*).

PINK1 was recently shown to directly phosphorylate Parkin on serine 65 at the Parkin N terminus, which may serve to activate Parkin (22). We therefore investigated the impact of this critical PINK1 phosphorylation site in Parkin on Miro ubiquitination levels upon FCCP-induced mitochondrial damage in HeLa cells (which do not express endogenous Parkin (45)) or in SH-SY5Y cells (Fig. 6, *D* and *E,* respectively), expressing wild type Parkin or Parkin containing either phospho-null or phospho-mimetic mutations at Ser-65 (S65A or S65E). In contrast to the robust FCCP-induced ubiquitination of ^myc^Miro1 observed in HeLa cells and SH-SY5Y cells expressing wild type ^YFP^Parkin, Miro1 ubiquitination was greatly reduced when ^YFP^Parkin^S65A^ was expressed, supporting a key role for PINK1-dependent phosphorylation of Parkin in Miro1 ubiquitination. Interestingly, in cells expressing the S65E Parkin mutant, Miro expression levels appeared substantially reduced under basal conditions suggesting constitutive activity of the phospho-mimetic S65E Parkin mutant to regulate Miro, potentially due to impaired Ubl domain-mediated inhibition of the ligase (Fig. 6, *D* and *E*) (46). In Parkin S65E-overexpressing cells, mitochondrial damage induced some further Miro loss and ubiquitination suggesting that additional regulatory mechanisms may further contribute to mitochondrial damage-induced activation of Parkin.

**FIGURE 7.**
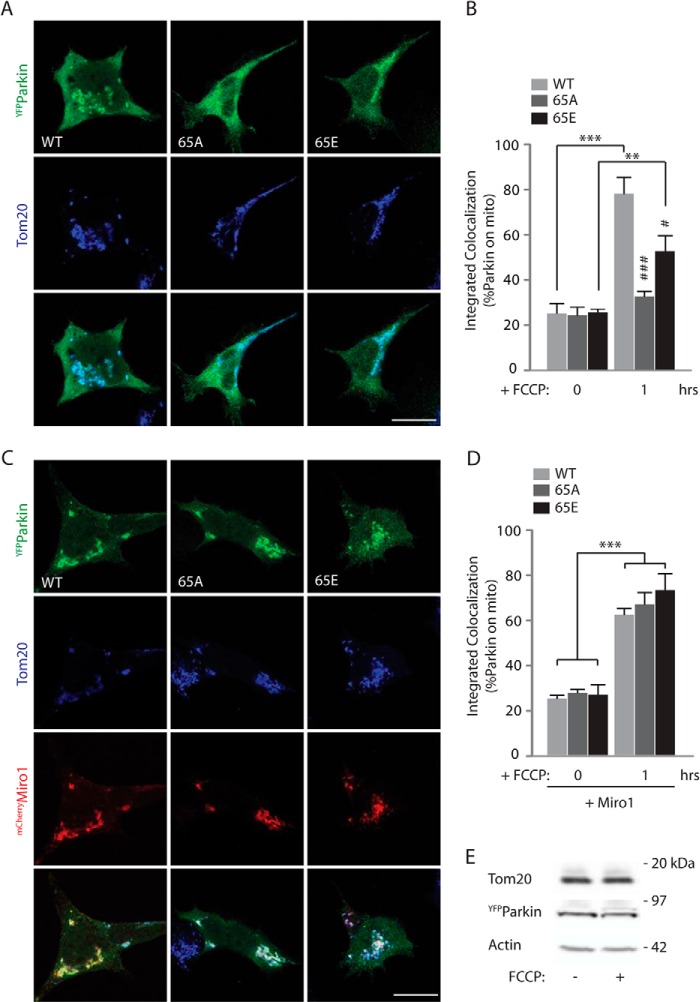
**Miro1 facilitates the recruitment of Parkin S65A and S65E to damaged mitochondria.** *A,* confocal images of HEK cells transfected with wild type ^YFP^Parkin or S65A or S65E Parkin mutants and treated with 10 μm FCCP for 1 h. *Scale bar*, 20 μm. *B,* Parkin recruitment to the mitochondria is analyzed as integrated colocalization of ^YFP^Parkin over the mitochondrial marker Tom20 and quantified in the *bar graph*. Percentage of wild type Parkin on mitochondria is 78.19 ± 4.86%; S65A Parkin is 33.27 ± 1.46%; S65E Parkin is 52.97 ± 4.68%; *n* = 9–14, #, *p* < 0.05; ###, *p* < 0.005. * is used to indicate significance between treated/untreated samples, whereas # is used to indicate significance within a treatment group, #, *p* < 0.05, **, *p* < 0.01; ###, ***p < 0.005. Data are shown as mean ± S.E. *C,* overexpression of ^mCherry^Miro1 together with ^YFP^Parkin or the S65A and S65E mutants facilitates Parkin recruitment to the mitochondria after 1 h of FCCP treatment. *Scale bar*, 20 μm. *D,* quantification of this effect. Percentage of wild type Parkin on mitochondria at 1 h when Miro1 is overexpressed at 61.69 ± 2.55%; S65A Parkin is 63.55 ± 5.24%; S65E Parkin is 72.82 ± 4.85%; *n* = 12–14, ***, *p* < 0.005. Data are shown as mean ± S.E. *E*, western blot showing that the mitochondrial marker Tom20 levels are not altered after 1 h of FCCP treatment (10 μm).

We also investigated whether the Ser-65 phospho-null and phospho-mimetic Parkin mutants were recruited in a similar way to wild type Parkin to damaged mitochondria and whether Miro had a role in regulating their translocation and stabilization on the OMM. Following FCCP treatment, nearly 80% of wild type Parkin was recruited to the damaged mitochondria, labeled with the mitochondrial marker Tom20, of which expression levels were not altered after 1 h of FCCP treatment (Fig. 7*E*). However, we found that the S65A and S65E Parkin mutants showed delayed recruitment dynamics. At early time points (1 h), we found a significant reduction in the translocation of the S65A and S65E mutants onto mitochondria compared with wild type Parkin (Fig. 7, *A* and *B*). However, 2 h after mitochondrial damage, both mutants were found to be translocated on the mitochondria to the same extent as wild type Parkin (data not shown), indicating that the translocation of these Parkin mutants was delayed compared with that of wild type Parkin as recently reported (47). Interestingly, we found that overexpressing wild type ^mCherry^Miro1 significantly increased the recruitment and stabilization of both the S65A and S65E mutants at early time points (Fig. 7, *C* and *D*). This suggests that upon mitochondrial damage Miro1 may act as a component of a Parkin receptor complex for activated Parkin on the OMM.

##### Impact of Parkin Ser-65 Mutants on Miro Degradation in Primary Neurons

**FIGURE 8.**
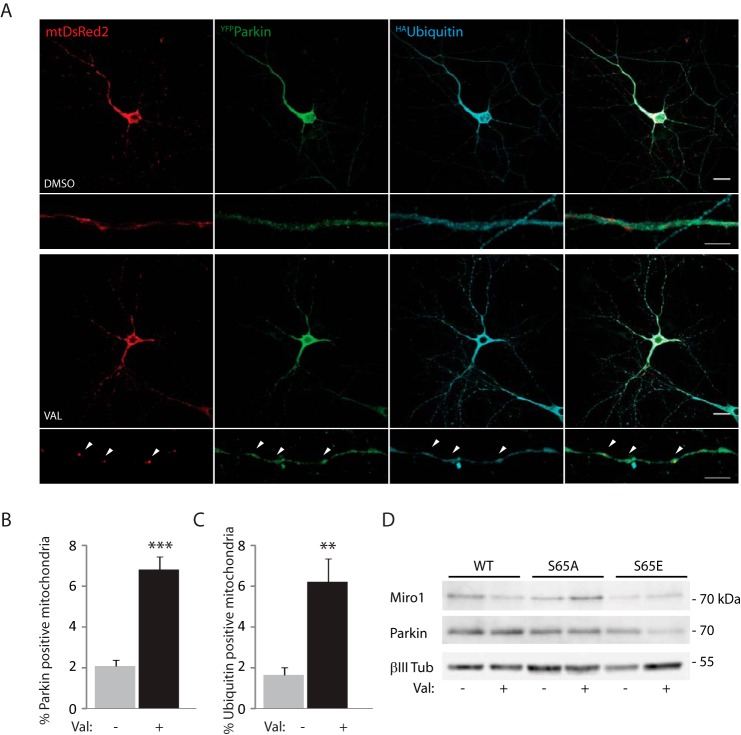
**Parkin is recruited to damaged mitochondria in neurons and determines Miro1 loss.** *A,* confocal imaging of hippocampal neurons overexpressing ^YFP^Parkin, ^HA^ubiquitin, and the mitochondrial marker mtDsRed2 shows that valinomycin (*Val*) treatment (2 μm, 90 min) of the neuronal culture induces Parkin translocation to mitochondria and ubiquitination of the organelles (*arrowheads*; *scale bar,* 20 or 5 μm for the zoomed image). *B* and *C, bar graphs* quantifying, respectively, Parkin recruitment and ubiquitin-positive mitochondria (6.83 ± 0.63% of total mitochondria were Parkin-positive after 90 min of valinomycin compared with 2.08 ± 0.32% Parkin-positive mitochondria with DMSO control; ***, *p* < 0.001; 6.21 ± 1.14% of total mitochondria were ubiquitin-positive after 90 min of valinomycin treatment, compared with 1.65 ± 0.37% with DMSO control, **, *p* < 0.005; *n* = 3, 3 cells per *n*). Data are shown as mean ± S.E. *D,* mitochondrial damage (valinomycin, 2 μm, 90 min) triggers the loss of immunopurified ^myc^Miro1 in cortical neurons overexpressing wild type Parkin but not S65A or S65E Parkin; *n* = 3.

Our results further support a critical role for phosphorylation at Ser-65 in Parkin for regulating substrate ubiquitination of OMM proteins and mitophagy. To assess mitophagy and investigate the role of the Ser-65 within Parkin in primary neurons, rat hippocampal cells were transfected with ^YFP^Parkin and ^HA^ubiquitin together with the mitochondrial marker mtDsRed2. Mitochondrial damage triggered by valinomycin (2 μm, 90 min), a mitochondrial uncoupling agent (potassium ionophore) that induces, in a similar fashion to FCCP, PINK1-dependent Parkin phosphorylation (22), led to a robust translocation of ^YFP^Parkin to mitochondria that correlates with an increased and localized ^HA^ubiquitin signal on the organelles (Fig. 8, *A–C*).

Finally, to further investigate the role of the Parkin Ser-65 site in regulating substrate ubiquitination during mitophagy in primary neuronal cells, we performed biochemical experiments to assess Miro1 loss in rat cortical neurons transfected with either wild type ^YFP^Parkin or S65A and S65E Parkin mutants. In neurons expressing wild type ^YFP^Parkin and ^myc^Miro1, a robust loss of Miro1 could be detected upon valinomycin-induced mitochondrial damage, similar to what we observed in SH-SY5Y dopaminergic cells. In contrast, in neurons expressing S65A or S65E Parkin mutants, we did not observe a significant damage-induced decrease of Miro1 levels (Fig. 8*D*).

## DISCUSSION

Here, we characterize in dopaminergic SH-SY5Y neuroblastoma cells, PD patient-derived fibroblasts, and primary rat neurons the mitochondrial damage-induced turnover and ubiquitination dynamics of the Miro mitochondrial trafficking complex. We find that FCCP-induced mitochondrial damage drives a robust loss of Miro at its expected molecular weight dependent on PINK1 and Parkin, and this is disrupted in PD patient-derived fibroblasts (lacking functional Parkin). Miro loss correlates with its fast ubiquitination on the OMM where it remains stably ubiquitinated prior to degradation. Using phospho-mimetic and phospho-null mutants, we also find that Ser-65 within Parkin, which is a substrate of PINK1-mediated phosphorylation, acts as a key point of regulation of the pathway. Moreover, we demonstrate the key role of the Parkin Ser-65 residue for regulating mitochondrial damage-induced ubiquitination of Miro1 in primary neurons.

Although our results are consistent with the PINK1/Parkin pathway targeting Miro to block mitochondrial trafficking, our results do not support a rapid, mitochondrial damage-induced depletion of Miro from the OMM. The loss of Miro at its expected molecular weight upon mitochondrial damage reported here and in earlier reports (18, 19) is primarily due to a shift from the expected molecular weight of Miro to higher molecular weight species upon ubiquitination. Consistent with this, we found that Miro1 was robustly ubiquitinated in dopaminergic SH-SY5Y cells upon mitochondrial depolarization similar to previous observations in HeLa cells and *Drosophila* neurons (19). Our analysis of the kinetics of Miro ubiquitination does not, however, support a mechanism whereby Miro ubiquitination leads to its fast degradation to underpin a rapid uncoupling of mitochondria from the kinesin transport pathway and the consequent mitochondrial arrest. Moreover, in our system, the interaction between Miro1 and the kinesin adaptor TRAK1 is not affected by mitochondrial damage. Rather, our results suggest a model whereby Miro is initially rapidly ubiquitinated on the OMM where it remains localized in a ubiquitinated form for some considerable time prior to it being turned over, dependent on the activity of the proteasome. This suggests that Miro ubiquitination (rather than ubiquitination-induced Miro degradation) may directly act as a rapid signal on the OMM for mitochondrial arrest. A number of mechanisms could underpin this. For example, ubiquitinated Miro could mediate the recruitment of other factors such as p62 and/or HDAC6 to the mitochondrial trafficking complex to facilitate the clustering of damaged mitochondria prior to their clearance by mitophagy. Moreover, Miro ubiquitination may also either directly or, by recruitment of HDAC6 and/or p62, sterically hinder its association or functional interaction with microtubule motor proteins leading to mitochondrial arrest.

We find Miro1 ubiquitination to include atypical Lys-27-linked ubiquitin chains, which have so far only been observed in connection with Parkin (13). It is interesting that modification with this chain type does not seem to lead to Miro1 immediate degradation, which is consistent with previous proteomic analysis showing that Lys-27 chains are not significantly enriched when the proteasome is inhibited, in contrast to Lys-48 or Lys-11 chains (48). This suggests that the chain type may have other more specific roles such as mediating mitophagy. Interestingly, Lys-27-linked polyubiquitin chains have previously been correlated to p62 translocation to damaged mitochondria (13).

We also provide new insights into the regulation of Miro turnover and ubiquitination dynamics by PINK1-dependent phosphorylation, which remains controversial. An initial report demonstrated robust phosphorylation of Miro at Ser-156 by PINK1 *in vitro* (18), although a more recent study was unable to find phosphorylation of Miro *in vitro* or a substantial role for the phosphorylation of these sites in the regulation of Miro stability (19). In our experiments, we did not find that the Miro1 Ser-156 phosphorylation site played a critical role in Miro1 ubiquitination or turnover in dopaminergic SH-SY5Y neuroblastoma cells. Although Miro phosphorylation by PINK1 could impact the rate or kinetics of Miro ubiquitination by Parkin, importantly, we did not find any substantial difference in the ubiquitination kinetics of phospho-null Miro1 S156A compared with wild type Miro1. Thus, phosphorylation of Miro1 by PINK1 to regulate Miro stability and ubiquitination may occur in a cell-specific manner or have a cell-specific impact on Miro ubiquitination kinetics, depending on the expression levels of Parkin or of other regulatory factors such as Miro protein phosphatases. In agreement with our data, in a recent screen of 16 putative PINK1 substrates (including Miro2), only Parkin was found to be robustly phosphorylated by PINK1 *in vitro* (22).

In contrast, we found that Ser-65 in Parkin, recently reported to be robustly phosphorylated by PINK1 (22, 47), played a critical role in regulating Miro ubiquitination levels. In both HeLa cells and SH-SY5Y neuroblastoma cells, expression of phospho-null S65A Parkin leads to substantially reduced Miro ubiquination (although Parkin S65A was translocated onto the mitochondria in these conditions) that correlates with an increased stability of the protein. The S65E phospho-mimetic Parkin had instead the opposite effect, apparently increasing Miro turnover under basal conditions. A slight residual ubiquitination of Miro1 could be detected when Parkin S65A was expressed, suggesting that other E3 ubiquitin ligases could act together with Parkin to ubiquitinate Miro. We also investigated the role of Ser-65 in primary cultured neurons. We show that as in HeLa cells and SH-SY5Y cells, Miro stability is regulated by this key site within Parkin. Parkin activation is tightly tuned and requires several levels of activation. Following recruitment to damaged mitochondria, Parkin needs to be activated by phosphorylation on its Ser-65 residue. As recently suggested (22, 46, 47), modifying the charge of the Ubl domain of Parkin (by mutagenesis or phosphorylation) could release its autoinhibition, favoring an active conformation of the E3 ubiquitin ligase. S65E Parkin mutant could therefore more easily interact with and modify its substrates leading to their increased turnover and decreased steady state levels.

Interestingly, we also found that Miro levels on the mitochondria could stabilize the phospho-null (S65A) and phospho-mimetic (S65E) versions of Parkin on the mitochondria in HEK cells. These results suggest that the levels of Miro (and potentially other OMM Parkin substrates as recently suggested for Mfn2 (49)) may play an additional role in stabilizing Parkin on the OMM. This would suggest that substrate binding by Parkin in conjunction with its phosphorylation could act together to stabilize Parkin on the OMM, providing a mechanism to tune Parkin levels to that of substrates on the OMM.

These findings provide an in depth analysis of Miro1 dynamics following mitochondrial damage highlighting its implication in mitophagy. Moreover, we uncover a role of Miro as part of the Parkin receptor complex on the OMM and its potential role in tuning Parkin-mediated mitochondrial quality control. Our data corroborate an important role of Lys-27-linked polyubiquitin in this process. We also disclose the importance of Ser-65 within Parkin as a regulator of its function and stability on damaged mitochondria. This may open new strategies to target mitochondrial dysfunction in PD pathogenesis.

## Footnotes

8 The abbreviations used are:

OMM: outer mitochondrial membrane
PD: Parkinson disease
FCCP: carbonyl cyanide *p*-trifluoromethoxyphenylhydrazone
DUB: deubiquitinase
OE: overexpressing.
